# Effect of *β-1,4-GalTI* on the biological function of astrocytes treated by LPS

**DOI:** 10.17305/bb.2024.11088

**Published:** 2024-09-13

**Authors:** Jiyu Li, Hui Jin, Xinmin Zhao, Xinran Sun, Jiyuan Zhong, Jian Zhao, Meijuan Yan

**Affiliations:** 1 Department of Orthopedic Oncology, Second Affiliated Hospital of Naval Medical University, Shanghai, China; 2 The Jiangsu Key Laboratory of Neuroregeneration, Nantong University, Nantong, China

**Keywords:** β-1,4-galactosyltransferase I, lipopolysaccharide (LPS), cell proliferation and migration, astrocytes

## Abstract

Inflammation of the central nervous system (CNS) is a common feature of neurological disorders and infections, playing a crucial role in the development of CNS-related conditions. CNS inflammation is primarily regulated by glial cells, with astrocytes being the most abundant type in the mammalian CNS. Numerous studies have demonstrated that astrocytes, as immunocompetent cells, perform diverse and complex functions in both health and disease. Glycosylation, a critical post-translational modification of proteins, regulates numerous biological functions. The expression and activity of glycosyltransferases, the enzymes responsible for glycosylation, are closely associated with the pathogenesis of various diseases. *β-1,4-GalTI,* a mammalian glycosyltransferase, plays a significant role in cell–cell interactions, adhesion, and migration. Although many studies have focused on *β-1,4-GalTI,* few have explored its effects on astrocyte function. In this study, we constructed lentiviral vectors for both interference and overexpression of *β-1,4-GalTI* and discovered that *β-1,4-GalTI* knockdown inhibited astrocyte migration and proliferation, while its overexpression promoted these processes. Concurrently, *β-1,4-GalTI* knockdown reduced the expression of tumor necrosis factor-α, interleukin-1β, and interleukin-6, whereas overexpression enhanced the expression of these cytokines. These findings suggest that modulating *β-1,4-GalTI* activity can influence the molecular functions of astrocytes and provide a theoretical foundation for further research into *β-1,4-GalTI* as a potential therapeutic target in astrocyte-mediated inflammation.

## Introduction

Central nervous system (CNS) inflammation is mainly regulated by glial cells. Astrocytes are the predominant type of glial cells in the mammalian CNS [1]. They play a variety of complex functions in diseases and inflammation [2–6]. Astrocytes exhibit both anti-inflammatory and pro-inflammatory roles, suggesting that their functions may vary across different CNS disorders. Even within the same disease astrocytes may, due to the time course of pathogenic mechanisms, their location in the CNS, and simultaneous exposure to local or peripheral pathogen-associated molecular patterns (PAMPs) and different cellular factors, play diverse roles in biological processes [7]. When the CNS is damaged, astrocytes can directly sense injury stimuli and undergo a series of biological changes, including transitioning from a normal state to a reactive state through gliosis and the secretion of cytokines and chemokines in response to various forms of CNS damage [8, 9], which has become a pathological feature of CNS structural damage [10]. With the comprehensive and in-depth study of astrocyte function, astrocytes are expected to become potential targets for the treatment of neuroinflammation-related diseases [11].

Glycosylation is one of the most significant post-translational modifications of proteins in organisms, regulating a wide range of biological functions [12]. Glycosyltransferases, the enzymes responsible for glycosylation, are a class of enzymes predominantly located in the endoplasmic reticulum and Golgi apparatus [13]. The *β-1,4-glycosidically* linked galactosyltransferase is one of the most studied glycosyltransferases in recent years. There are seven members in this family, sharing 25%–55% sequence homology but differing in substrate specificities, tissue distribution, and biological functions [14]. *β-1,4-GalTI* was the first mammalian glycosyltransferase to be cloned and had its crystal structure solved first [15]. According to their homology with *β-1,4-GalTI,* they were named *β-1,4-GalTI* to *β-1,4-GalTVII* in turn. *β-1,4-GalTI* is a type II transmembrane glycoprotein comprising approximately 400 amino acids [16]. It features a short amino-terminal cytoplasmic domain, a transmembrane domain, and a large carboxy-terminal catalytic domain [17]. This enzyme catalyzes the transfer of galactose from uridine diphosphate-galactose (UDP-Gal) to the terminal N-acetylglucosamine (GlcNAc) on oligosaccharide chains of membrane-bound and secretory glycoconjugates [18]. *β-1,4-GalTI* is implicated in a variety of biological functions, including tumor development [19], neurite extension [20], and embryonic development [21]. Additionally, *β-1,4-GalTI* plays a crucial role in the onset and maintenance of CNS inflammatory responses [22].

Lipopolysaccharide (LPS) is a component of the outer membrane of Gram-negative bacteria, that interacts with and induces CNS inflammation in a variety of host cells, including astrocytes and microglia [23]. LPS-stimulated astrocytes can induce the production of various cytokines [14], including tumor necrosis factor-α (TNF-α), interleukin-1β (IL-1β), and interleukin-6 (IL-6). Cytokines TNF-α, IL-6, and IL-1β have been implicated in the pathogenesis of many CNS inflammation-related diseases, including TBI, AD, PD, and MS [24, 25]. IL-1β is a potent pro-inflammatory cytokine produced and secreted by activated monocytes, macrophages, and glial cells. It stimulates cyclooxygenase activity, leading to the release of prostaglandins, which are crucial for host defense mechanisms against injury and infection [26]. IL-6, another multifunctional cytokine, is produced by both lymphoid and non-lymphoid cells and serves as a key mediator in the CNS [27]. This cytokine is rapidly and transiently produced in response to injury and infection, promoting host defense by stimulating acute-phase responses, hematopoiesis, and immune reactions. IL-6 not only plays a critical role in mediating acute inflammatory responses but is also essential for maintaining normal brain functions. TNF-α is a crucial cytokine in inflammation [28]. It promotes the production of nitric oxide, IL-1β, and IL-6 and enhances the adhesion and permeability of endothelial cells. Furthermore, TNF-α facilitates the migration and recruitment of immune cells to inflammation sites [29]. Additionally, it plays significant roles in apoptosis, angiogenesis, tumorigenesis, CNS inflammation, and the onset of cognitive impairments following infection [30].

Numerous studies have demonstrated that *β-1,4-GalTI* plays a critical role in mediating secondary injury-induced inflammation and cytokine production following spinal cord injury [18]. Knockout of the *β-1,4-GalTI* gene in mice inhibited acute-phase inflammation, chronic inflammation, and the migration of inflammatory cells to the site of inflammation. In addition, *β-1,4-GalTI* present on the cell membrane plays a role in various cell adhesion, proliferation, and migration processes [15]. The substrate of Src-inhibited protein kinase C affects astrocyte migration and galactosylation of integrin β1 by functionally modulating *β-1,4-GalTI* catalysis in LPS-induced astrocyte inflammation. Although there are many studies on *β-1,4-GalTI,* there are few reports on the effect of *β-1,4-GalTI* on astrocyte function and its potential role in modulating neuroinflammation and neuroprotection. Therefore, we constructed an LPS-induced cellular inflammation model and used interference and overexpression strategies to explore the effect of *β-1,4-GalTI* intervention on the biological function of astrocytes.

## Materials and methods

### Construction of lentiviral expression vectors

The RNAi sequence targeting β-1,4-GalTI (GCACTGGATTGTTGACTCTGC) and a corresponding scrambled oligonucleotide sequence (TTCTCCGAACGTGTCACGT) were cloned into the AgeI/EcoRI sites of the hU6-MCS-Ubiquitin-EGFP-IRES-puromycin vector. The *β-1,4-GalTI* cDNA and its corresponding scrambled sequence were cloned into the BamHI/AgeI sites of the Ubi-MCS-3FLAG-CBh-gcGFP-IRES-puromycin plasmid. Lentiviral particles were produced in HEK-293T cells, and viral titers were quantified by Jikai Gene Chemical Technology, Shanghai, China. Commercially sourced reagents included: goat anti-*β-1,4-GalTI* antibody (1:400 for immunofluorescence and immunoblotting; Santa Cruz, CA, USA), rabbit anti-GFAP (1:400 for immunofluorescence; Abcam), goat anti-β-actin (1:2000 for immunoblotting; Cell Signaling Technology), Hoechst stain (1:4000 for immunofluorescence), and an EdU staining kit (Sigma-Aldrich).

### Animals

Adult male Sprague-Dawley rats weighing 200–250 g were obtained from the Experimental Animal Center of Nantong University, Nantong, China. Animal care, breeding, and experimental procedures were approved by the Animal Care and Use Committee of Nantong University (license number: S20190920-003) and the Animal Care Ethics Committee of Jiangsu Province. All procedures complied with internationally recognized guidelines for the care and use of laboratory animals.

### Primary astrocyte culture

Astrocyte cultures were established from 1-day-old Sprague-Dawley rats. After opening the skull, the brain was exposed, and the outer membrane of the cerebral cortex was removed. The cerebral cortex was dissected using a solution containing 99% PBS and 1% penicillin/streptomycin. The resultant mixture was centrifuged at 1200 rpm for 5 min. Enzymatic digestion was performed with 0.25% trypsin (Gibco-BRL) at 37 ^∘^C for 15 min. Subsequently, DMEM/F12 high glucose medium (Gibco) was added to terminate the enzymatic digestion. The filtrate, after passing through a 200-mesh sieve and centrifuging at 1200 rpm for 5 min, was collected. After a 72-h incubation at 37 ^∘^C with 5% CO_2_, the medium was replaced to remove any cell debris and non-adherent cells.

### Immunofluorescence

Astrocytes cultured on coverslips were fixed using 4% paraformaldehyde for 30 min at room temperature. After washing with PBS, cells were incubated with the primary antibody diluted in antibody diluent overnight at 4 ^∘^C. Cells were then washed with PBS and incubated with a Cy3-conjugated anti-rabbit or anti-mouse secondary antibody for 2 h at room temperature or alternatively, overnight at 4 ^∘^C. Subsequently, they were counterstained with Hoechst for 30 min, and the slides were mounted for imaging using a DMR inverted microscope (Leica DMi8, Germany).

### Identification of transfection efficiency

Astrocytes were cultured in Dulbecco’s Modified Eagle Media: Nutrient Mixture F-12 (DMEM/F12) medium (Gibco). The medium was supplemented with 10% FBS (Thermo Fisher Scientific) and 1% penicillin–streptomycin solution (Beyotime Biotechnology). Cultivation occurred in a controlled environment within a humidified incubator set a 37 ^∘^C with an atmosphere of 5% CO_2_ and 95% air. Cells were seeded in culture plates at a density of 1×10^5^ cells per square centimeter using DMEM/F12 medium supplemented with 10% FBS. After 12 h, the medium was replaced with fresh DMEM/F12 medium. Lentivirus particles tagged with EGFP were introduced to the cells according to the formula: Volume of lentivirus ═ multiplicity of infection × cell number/virus titer. After 72 h of transfection, the proportion of cells expressing EGFP was assessed by co-localization with cell immunofluorescence.

### Extraction of RNA and reverse-transcription polymerase chain reactions

Total RNA was isolated from the cerebral cortex using Trizol reagent (Sigma), following the manufacturer’s instructions. First-strand cDNA synthesis was performed using a reverse transcription kit (Vazyme) with an oligo (dT18) primer. Gene expression levels were quantified by quantitative reverse transcription polymerase chain reaction (qRT-PCR) using the LightCycler 96 system and SYBR Green PCR Master Mix (Vazyme), as per the manufacturer’s protocols. Gene-specific primers for rats, provided by Thermo Fisher Scientific, were used. β-actin was used as an internal control, and the experiments were replicated in three independent trials.

### Western blot analysis

Cells were lysed using an extraction buffer. Protein concentration was determined using a bicinchoninic acid (BCA) assay kit (Beyotime, Jiangsu, China). Protein extracts were heat-denatured by immersion in a boiling water bath for 5 min. Denatured proteins were then separated by sodium dodecyl sulfate-polyacrylamide gel electrophoresis (SDS-PAGE). Separated proteins were transferred onto a polyvinylidene fluoride (PVDF) membrane (Millipore, Bedford, MA, USA) for 120 min at 100 V. The PVDF membrane was blocked using 5% skim milk for 1 h. The membrane was then washed three times with Tris-buffered saline with Tween 20 (TBST) for 5 min each. Subsequently, the membrane was incubated with a *β-1,4-GalTI* goat polyclonal antibody (diluted 1:400 in Tris-buffered saline; Santa Cruz Biotechnology) overnight at 4 ^∘^C.

The membrane was washed three times with TBST for 15 min each, followed by incubation with a donkey anti-goat HRP-conjugated secondary antibody (1:1000; ab6885, Abcam) for 2 h at room temperature. HRP activity was detected using BeyoECL Star (Beyotime, Jiangsu, China) after washing the membrane. Images were scanned using a GS800 Densitometer (Bio-Rad), and the data were analyzed using PDQuest 7.2.0 software (Bio-Rad).

### EdU staining

Astrocytes were cultured in 96-well plates and treated with 1 µg/mL LPS for 24 h or left untreated. Proliferation was assessed using the Cell-Light EdU DNA cell proliferation kit (RiboBio, Guangzhou, China). Briefly, cells were incubated with a culture medium containing 50 µM EdU for 2 h. Cells were then fixed with 4% formaldehyde for 30 min. Following decolorization, samples were treated with a 2 mg/mL glycine solution for 5 min. Samples were then permeabilized with 0.5% Triton X-100 for 10 min. After washing thoroughly with PBS, cells were stained with Apollo dye for 30 min, followed by a 30-min Hoechst staining. Imaging was performed using a DMR inverted microscope (Leica DMi8, Germany). The proliferation index was calculated by dividing the number of EdU-positive cells by the total number of cells and multiplying by 100%.

### Cell counting kit-8 test

Astrocytes were cultured in 96-well plates and subsequently treated with either 1 µg/mL LPS or vehicle alone for 24 h. Proliferation was assessed using a CCK-8 kit (Dojindo Molecular Technologies, Kumamoto, Japan), according to the manufacturer’s instructions. Cells were incubated at 37 ^∘^C with 5% CO_2_ for 2 h. Subsequently, 10 µL of CCK-8 solution was added, and the assay was incubated for an additional 2 h to allow for color development. Cell growth was quantified by measuring absorbance at 450 nm using a microplate reader.

### Transwell^TM^ migration assay

A single-cell suspension of astrocytes from each group was prepared by collecting and resuspending them in DMEM/F12 medium at a concentration of 5×10^3^ cells/mL. The cell suspension (100 µL/well) was seeded onto the upper compartment of Transwell™ permeable supports with 8.0 µm pore polycarbonate membrane inserts (Costar, USA). These inserts were subsequently positioned in 24-well plates. In the lower chamber of the culture wells, 500 µL of cell culture medium containing 10% FBS was added. Following a 24-h incubation at 37 ^∘^C with 5% CO_2_, the Transwell™ inserts were carefully removed. The medium was aspirated from the inserts, and the Transwell™ inserts were fixed with 4% paraformaldehyde for 30 min at room temperature. Cells that migrated to the lower surface were stained with 0.1% crystal violet for 30 min. Remaining cells on the upper surface of the inserts were gently removed with a cotton swab. Imaging and statistical analysis were conducted using a DMR inverted microscope (Leica DMi8, Germany).

**Figure 1.**
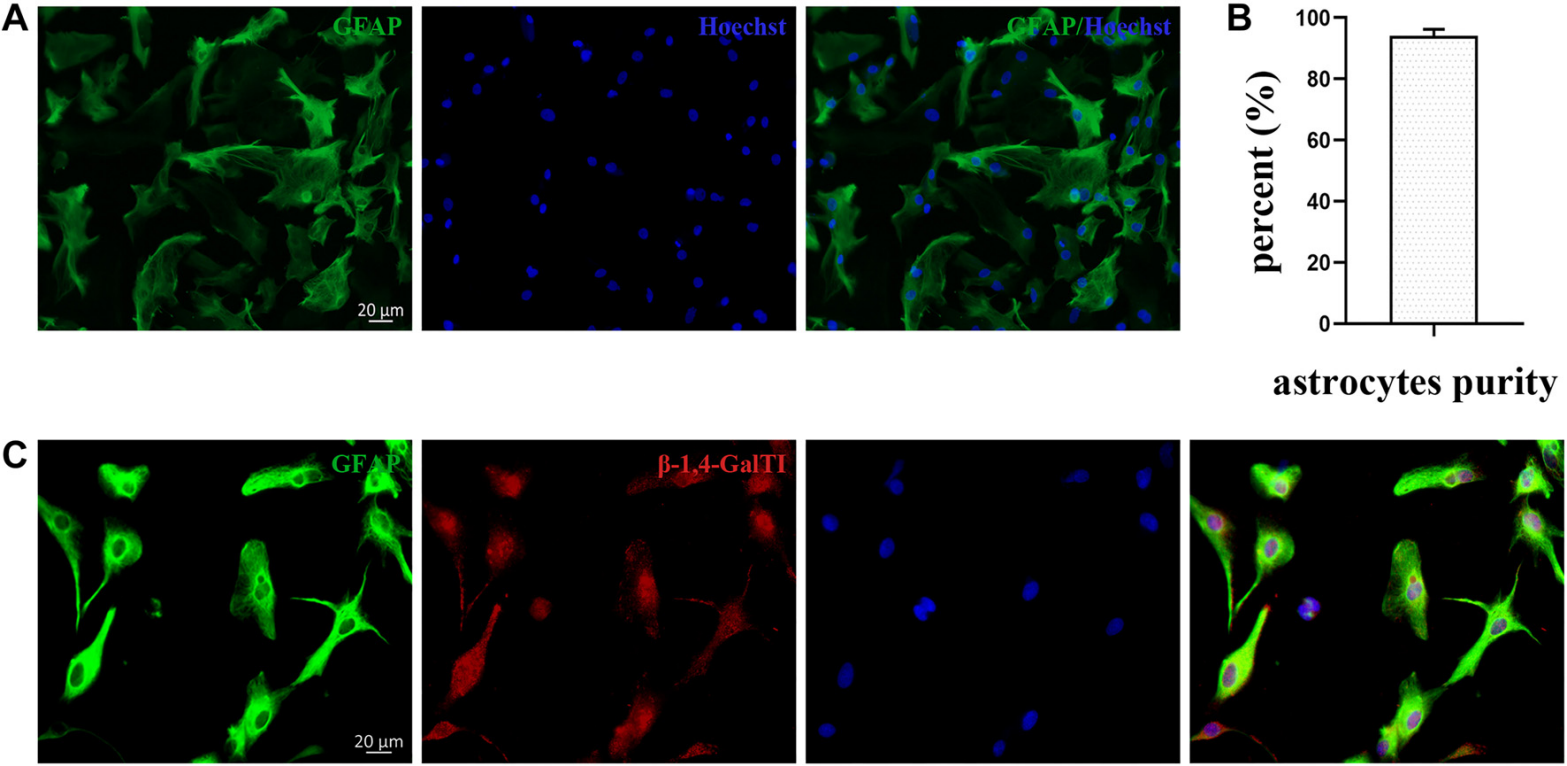
**Expression of *β-1,4-GalTI* in primary astrocytes.** (A) Immunofluorescence staining was used to assess the purity of primary astrocytes: GFAP (Green); Hoechst (Blue); scale bar ═ 20 µm; (B) Quantitative analysis of astrocyte purity; (C) Immunofluorescence staining was used to identify the expression of *β-1,4-GalTI* in primary astrocytes: GFAP (Green); *β-1,4-GalTI* (Red); Hoechst (Blue); scale bar ═ 20 µm.

### Scratch assay

Astrocytes were cultured in confluent layers in 6-well plates and mechanically injured using 200 µL pipette tips. Subsequently, the injured cells were rinsed with warmed DMEM/F12 medium, and the medium was replaced with 2 mL of fresh DMEM/F12 medium. The DMEM/F12 medium for LPS treatment was supplemented with 1 µg/mL LPS. Imaging was performed immediately and 24 h after wounding the cells. The migration index was calculated by measuring the ratio of the residual to the initial wound gap using ImageJ software (imagej.nih.gov/ij/)(NIH, America).

### Enzyme-linked immunosorbent assay (ELISA)

The cultured primary astrocyte supernatant was collected and centrifuged at 1000×*g* for 10 min to remove particles and aggregates. The concentrations of TNF-α, IL-1β, and IL-6 were quantified using ELISA kits from BD Biosciences and R&D Systems (Minneapolis, MN, USA). Absorbance was measured at wavelengths of 450 and 630 nm using a 96-well plate reader (Biotek Synergy2).

### Ethical statement

The animal study was reviewed and approved by the Animal Experiment Committee of Nantong University. The authors are accountable for all aspects of the work in ensuring that questions related to the accuracy or integrity of any part of the work are appropriately investigated and resolved. All animal care, breeding, and testing procedures were approved by the Animal Care and Use Committee of Nantong University (License number: S20190920-003) and the Animal Care Ethics Committee of Jiangsu Province, in compliance with internationally recognized and institutional guidelines for the care and use of animals. A protocol was prepared before the study without registration.

### Statistical analysis

Data are presented as mean ± standard deviation (SD). Statistical analyses were performed using one-way analysis of variance (ANOVA) and *t*-tests. Statistical significance was established at *P* < 0.05, while *P*> 0.05 indicated no significant difference. To ensure robust statistical analysis, we utilized software, such as GraphPad Prism for statistical calculations, and Adobe Photoshop and ImageJ (imagej.nih.gov/ij/) (NIH, America) for image processing. These results were obtained from three independent experiments.

## Results

### Expression of β*-1,4-GalTI* in primary astrocytes

To assess the presence of *β-1,4-GalTI* in cultured primary astrocytes from Sprague-Dawley rats, we first conducted a purity assessment of the cells. The findings demonstrated that the simultaneous presence of the astrocytic marker GFAP and the nuclear marker Hoechst33342 exceeded 90% (Figure 1), suggesting a significant level of astrocyte purity, appropriate for further investigation (Figure 1B).

We used cellular immunofluorescence double labeling to detect the co-localization of *β-1,4-GalTI* and the astrocyte marker GFAP in cultured SD rat primary astrocytes. These findings show that *β-1,4-GalTI* and GFAP are strongly co-localized in primary astrocytes, suggesting that *β-1,4-GalTI* is expressed in astrocytes. Hoechst nuclear staining also showed that *β-1,4-GalTI* was present in both the cell nucleus and the cytoplasm (Figure 1C).

### Screening and identification of effective lentiviral expression vectors

**Figure 2.**
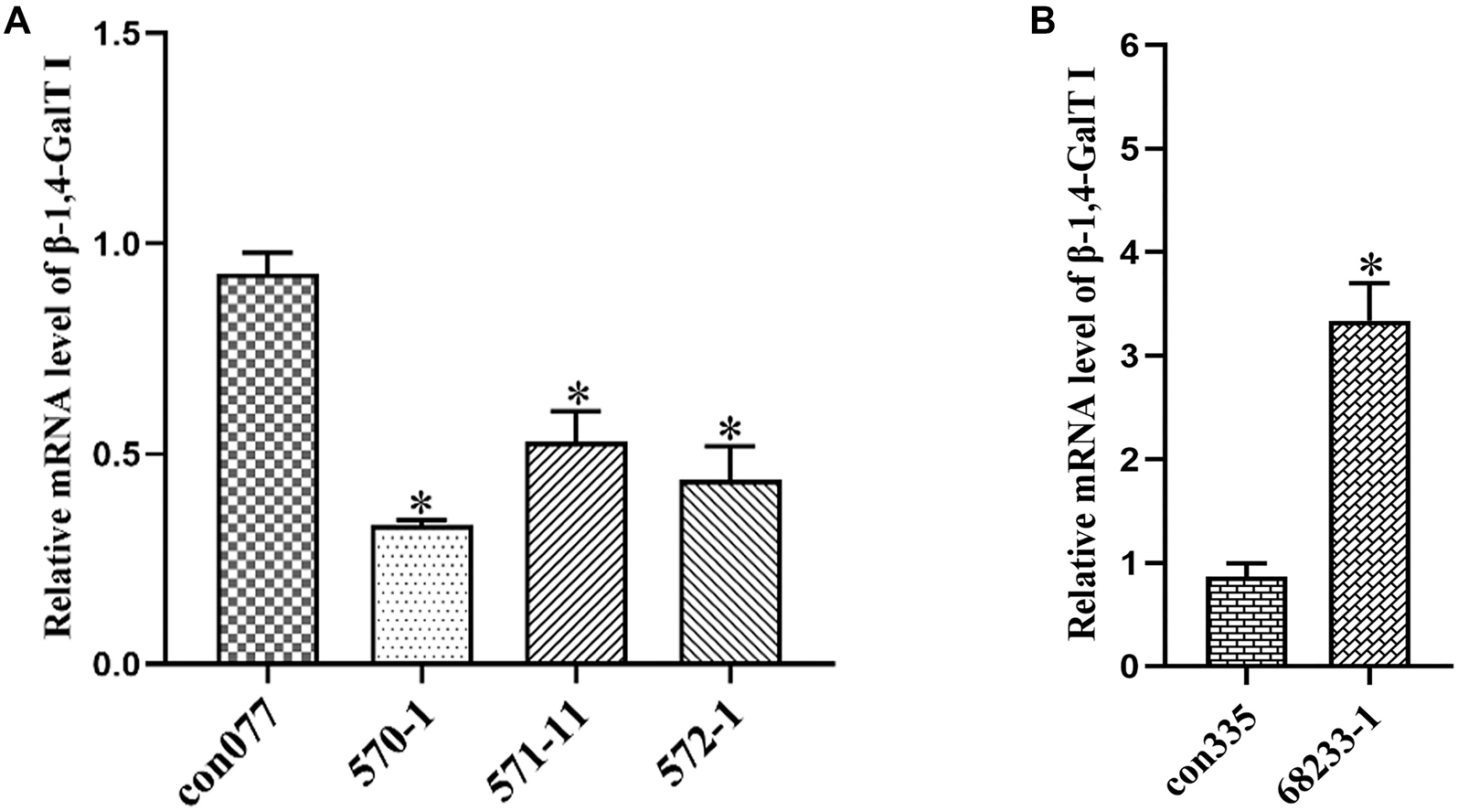
**Screening and identification of effective lentiviral expression vectors.** (A) qRT-PCR detection of the interference effect of *β-1,4-GalTI* on lentiviral expression vectors; con077, *β-1,4-GalTI* interferes with the lentiviral empty expression vector; 570-1, 571-11, and 572-1 are all *β-1,4-GalTI* interference lentiviral expression vectors, *n* ═ 3 per group; ^*^P < 0.05 vs con077; (B) qRT-PCR detection of the overexpression effect of *β-1,4-GalTI* overexpression lentiviral expression vector; con335, *β-1,4-GalTI* overexpressed lentiviral empty expression vector; 68233-1, *β-1,4-GalTI* overexpressed lentiviral expression vector, *n* ═ 3 per group; ^*^P < 0.05 vs con335. qRT-PCR: Quantitative reverse transcription polymerase chain reaction.

The examination involved the use of RT-PCR to analyze three lentiviral expression vectors (570-1, 571-11, and 572-1) that interfere with *β-1,4-GalTI,* as well as an empty vector con077. The results demonstrated that all three lentiviral expression vectors exhibited interference effects compared to the empty vector. The lentiviral vector labeled as 570-1 exhibited the highest level of interference among all the vectors (Figure 2A). Therefore, we selected the *β-1,4-GalTI* interfering lentiviral vector labeled as 570-1 for further investigation.

**Figure 3.**
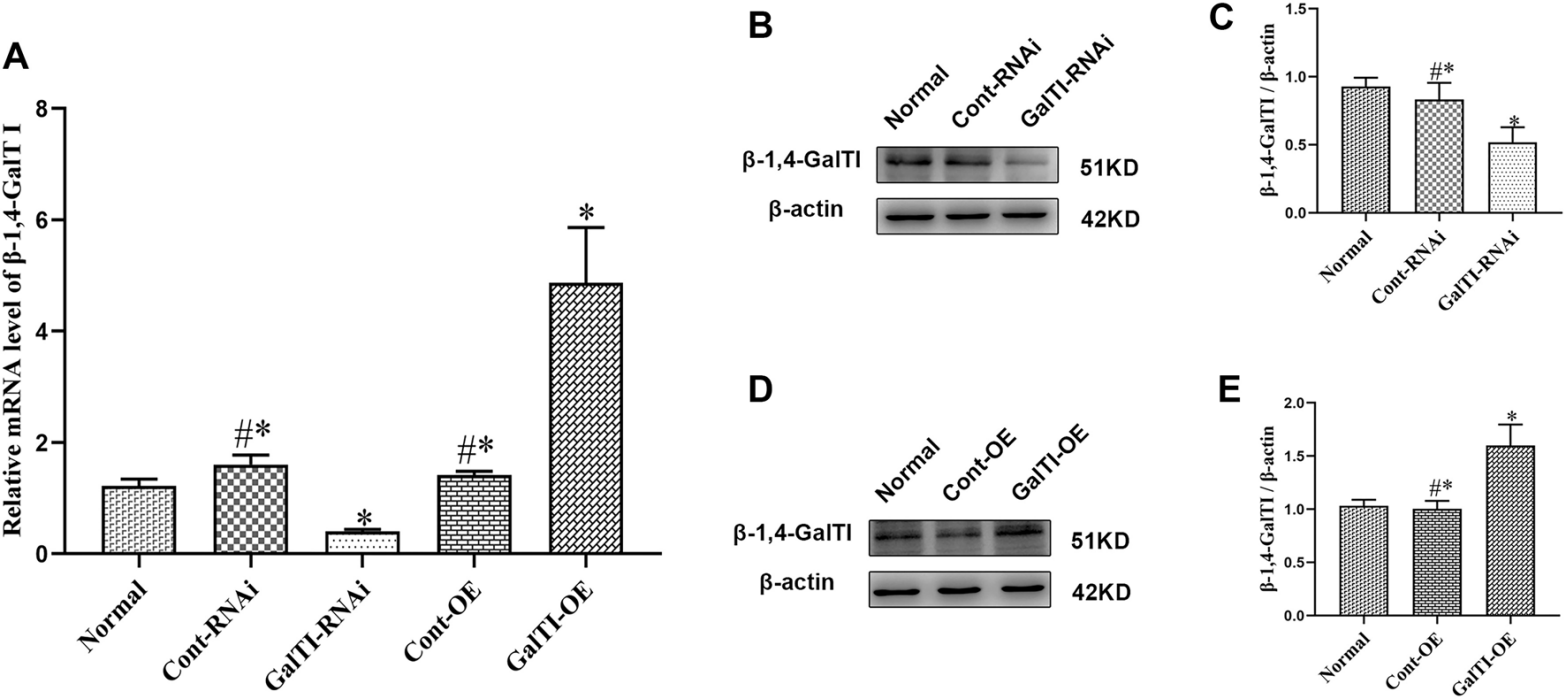
**The expression of *β-1,4-GalTI* in astrocytes.** (A) and (C) Detection of *β-1,4-GalTI* expression in astrocytes by Western blot cytometry; *n* ═ 3 per group, ^*^P < 0.05 vs Normal,^#*^P> 0.05 vs Normal; (B) and (D) Quantitative analysis of western blot results; (E) Detection of *β-1,4-GalTI* expression in astrocytes by qRT-PCR morphocytometry, *n* ═ 3 per group; ^*^P < 0.05 vs Normal,^#*^P> 0.05 vs Normal. qRT-PCR: Quantitative reverse transcription polymerase chain reaction.

The level of *β-1,4-GalTI* lentiviral expression vector 68233-1 was assessed using RT-PCR in comparison to the overexpression empty vector con335. The experimental results indicated that the *β-1,4-GalTI* lentiviral expression vector 68233-1 exhibited a more potent overexpression effect than the control empty vector con335. These findings indicate that the development of the *β-1,4-GalTI* lentiviral overexpression vector was successful and can be used for subsequent research (Figure 2B). Western blot analysis was employed to detect the expression of *β-1,4-GalTI* in astrocytes across different groups. The results from the one-way ANOVA revealed that the expression level of *β-1,4-GalTI* in the GalTI-RNAi group was significantly lower compared to the Normal group (Figure 3A and 3B) On the other hand, the GalTI-OE group had a higher expression (Figure 3C and 3D). Both differences were statistically significant, with *P* values less than 0.05. The Cont-RNAi group and the Cont-OE group exhibited no statistically significant differences compared to the Normal group (both *P*> 0.05), consistent with the PCR findings (Figure 3E).

**Figure 4.**
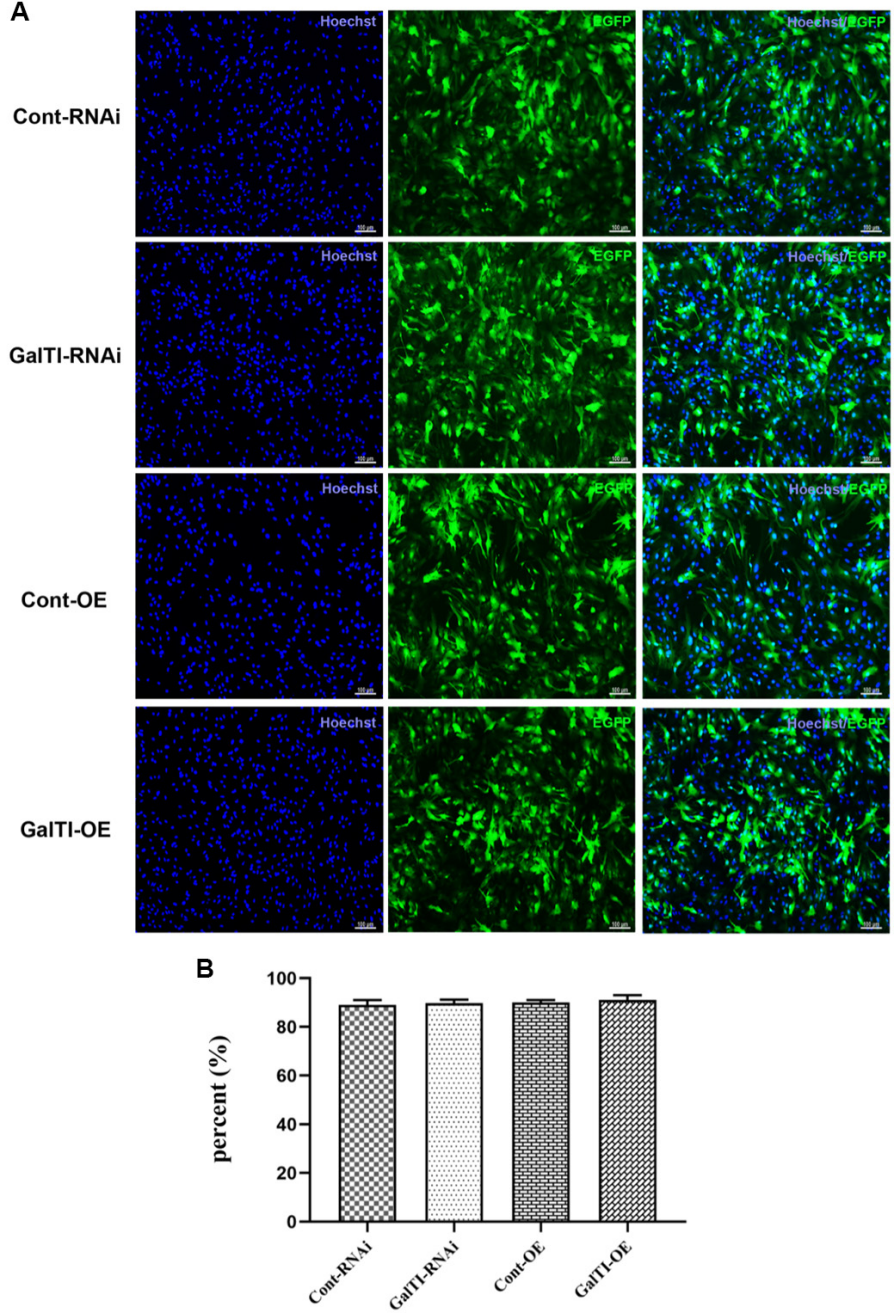
**Identification of transfection efficiency of disease lentiviral vector in astrocytes.** (A) Hoechst (blue); EGFP (lentiviral expression vector, green); Merge (combination of A and B); bar ═ 100 µm; (B) Quantitative analysis of transfection efficiency, *n* ═ 3 per group.

Subsequently, primary astrocytes were transfected with lentiviral vectors containing interfering sequences targeting *β-1,4-GalTI.* Additionally, primary astrocytes were transfected with lentiviral vectors including sequences that overexpress *β-1,4-GalTI.* Cellular immunofluorescence was employed after a period of 72 h to determine the transfection efficiency of each group of lentiviral vectors in astrocytes. The results showed that the transfection efficiency of the Cont-RNAi group (con077 group), GalTI-RNAi group (570-1 group), Cont-OE group (con335 group), and GalTI-OE group (68233-1) all exceeded 85%, demonstrating their appropriateness for further investigation (Figure 4).

### Effects of β*-1,4-GalTI* intervention on the proliferation of LPS-treated primary astrocytes

There is more cell division and movement among astrocytes near damaged areas in the CNS during injuries. This activity contributes to the formation of glial scars, which inhibit axonal regeneration [31–34]. Therefore, targeting the excessive proliferation and migration of astrocytes may provide a novel strategy for CNS repair. This work aimed to assess the effect of *β-1,4-GalTI* intervention on the proliferation of astrocytes following LPS treatment. We conducted EdU proliferation studies and CCK-8 assays to analyze this influence.

**Figure 5.**
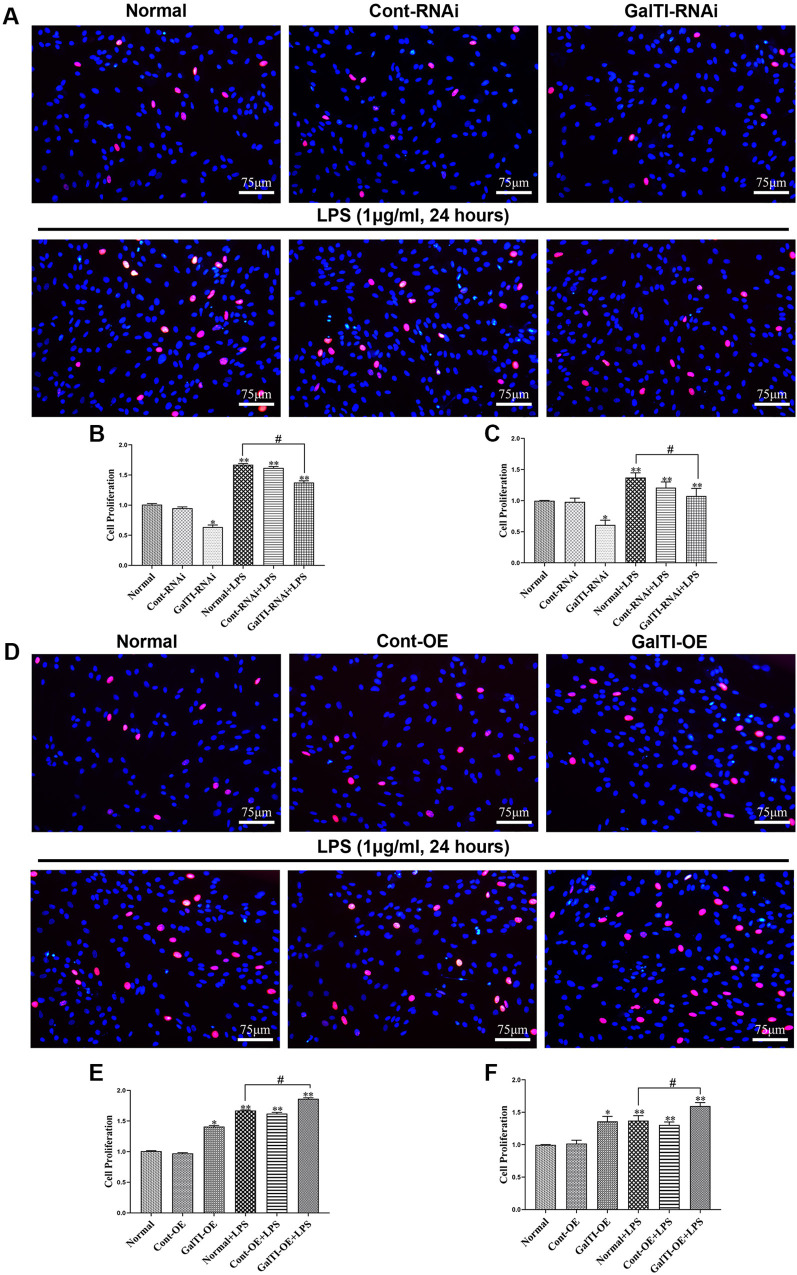
**Effects of *β-1,4-GalTI* intervention on the proliferation of LPS-treated primary astrocytes.** (A) EdU detects the effect of *β-1,4-GalTI* interference on astrocyte proliferation (red: proliferating cells, blue: Hoechst); (B) Quantitative analysis of EdU detects the effect of *β-1,4-GalTI* interference; (C) Quantitative analysis of CCK8 detects the effect of *β-1,4-GalTI* interference; (D) EdU detection of the effect of *β-1,4-GalTI* overexpression on astrocyte proliferation (red: proliferating cells, blue: Hoechst); (E) Quantitative analysis of EdU detects the effect of *β-1,4-GalTI* overexpression; (F) Quantitative analysis of CCK8 detects the effect of *β-1,4-GalTI* overexpression. *n* ═ 3 per group, ^*^P < 0.05 vs Normal,^**^P < 0.01 vs Normal/Cont-RNAi/GalTI-RNAi, ^**^P < 0.01 vs Normal/Cont-OE/GalTI-OE,^#^*P* < 0.05 vs Normal + LPS; bar ═75 µm. LPS: Lipopolysaccharide.

Results from the EdU proliferation experiment in the *β-1,4-GalTI* interference group revealed that, compared to the Normal, Cont-RNAi, and GalTI-RNAi groups, the proliferation capacity of astrocytes was significantly higher in the Normal+LPS, Cont-RNAi+LPS, and GalTI-RNAi+LPS groups (all *P* < 0.05), indicating that LPS stimulates astrocyte proliferation. In contrast, the GalTI-RNAi group demonstrated a significant reduction in astrocyte proliferation compared to the Normal group (*P* < 0.05). Similarly, the GalTI-RNAi+LPS group showed decreased proliferation relative to the Normal+LPS group (*P* < 0.05). These findings suggest that interference with *β-1,4-GalTI* effectively inhibits astrocyte proliferation (Figure 5A and 5B). At the same time, the results of the EdU proliferation experiment indicated that astrocytes with overexpression of *β-1,4-GalTI* showed a significantly increased proliferation capacity compared to the Normal, Cont-OE, and GalTI-OE groups. Additionally, the Normal+LPS, Cont-OE+LPS, and GalTI-OE+LPS groups demonstrated enhanced astrocyte proliferation (all *P* < 0.05), suggesting that LPS actively promotes astrocyte proliferation. Furthermore, the GalTI-OE group exhibited a higher proliferation rate than the Normal group (*P* < 0.05). Similarly, astrocyte proliferation in the GalTI-OE+LPS group was significantly greater than in the Normal+LPS group (*P* < 0.05). These findings indicate that overexpression of *β-1,4-GalTI* effectively stimulates astrocyte proliferation (Figure 5D and 5E). The results of CCK-8 detection are consistent with the EdU results (Figure 5C and 5F).

### Effects of β*-1,4-GalTI* intervention on the migration of LPS-treated primary astrocytes

Previous research has indicated that during the LPS-induced inflammatory response in the CNS, *β-1,4-GalTI* and the Galβ1-4GlcNAc sugar chains it catalyzes may play a crucial role in the migration of inflammatory cells to the site of inflammation [18]. Additionally, substrates of Src-inhibited protein kinase C can enhance LPS-sensitized astrocyte migration [35]. In this study, we investigated the migration of LPS-sensitized astrocytes following intervention with *β-1,4-GalTI* using both Transwell migration experiments and scratch assays.

**Figure 6.**
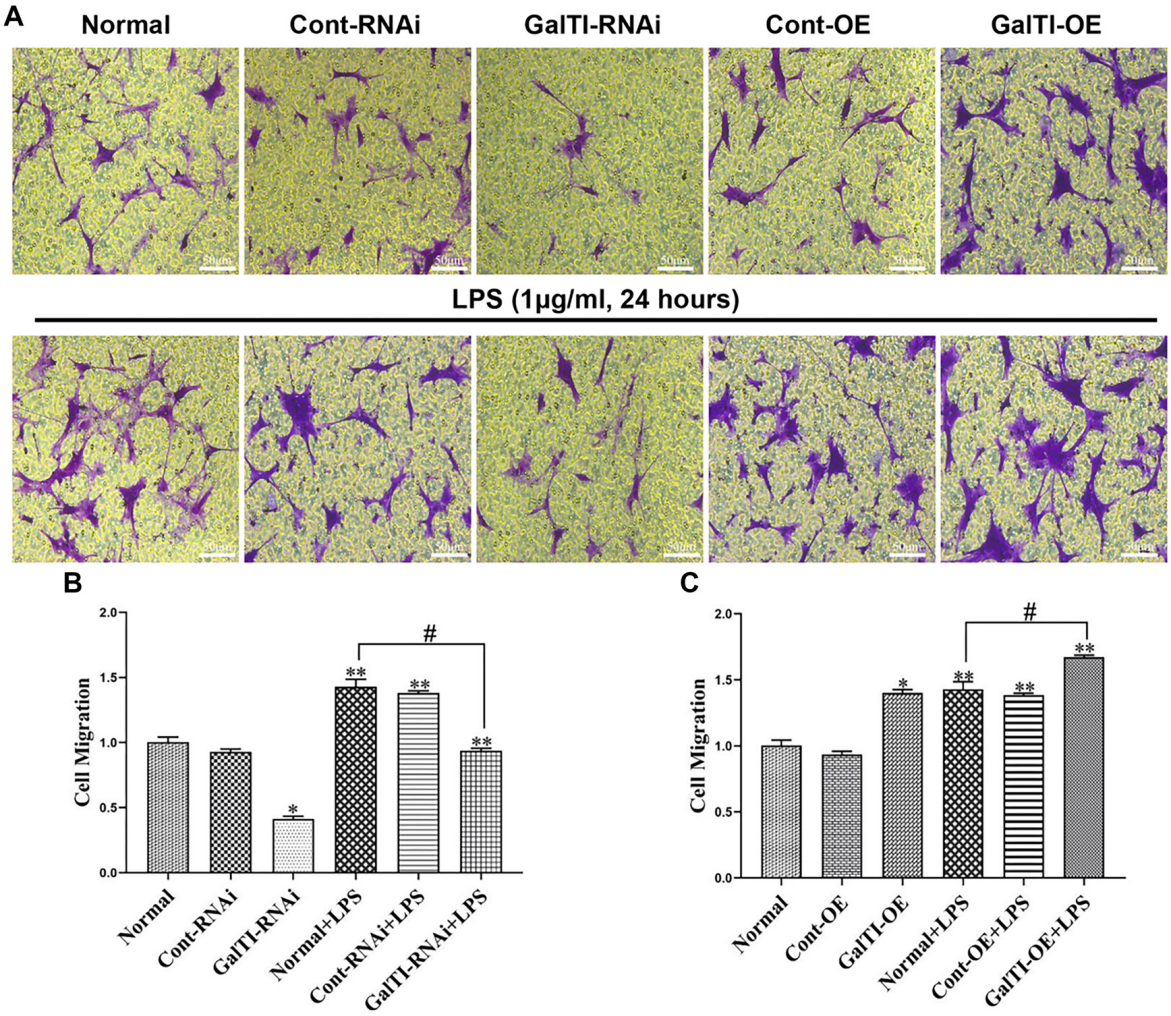
**Effects of *β-1,4-GalTI* intervention on the migration of LPS-treated primary astrocytes.** (A) Migration of astrocytes in each group; (B) and (C) Quantitative analysis of Transwell migration experiments; *n* ═ 3 per group, ^*^P < 0.05 vs Normal,^**^P < 0.01 vs Normal/Cont-RNAi/GalTI-RNAi/Cont-OE/GalTI-OE, ^#^*P* < 0.05 vs Normal+LPS; Bar ═ 50 µm. LPS: Lipopolysaccharide.

**Figure 7.**
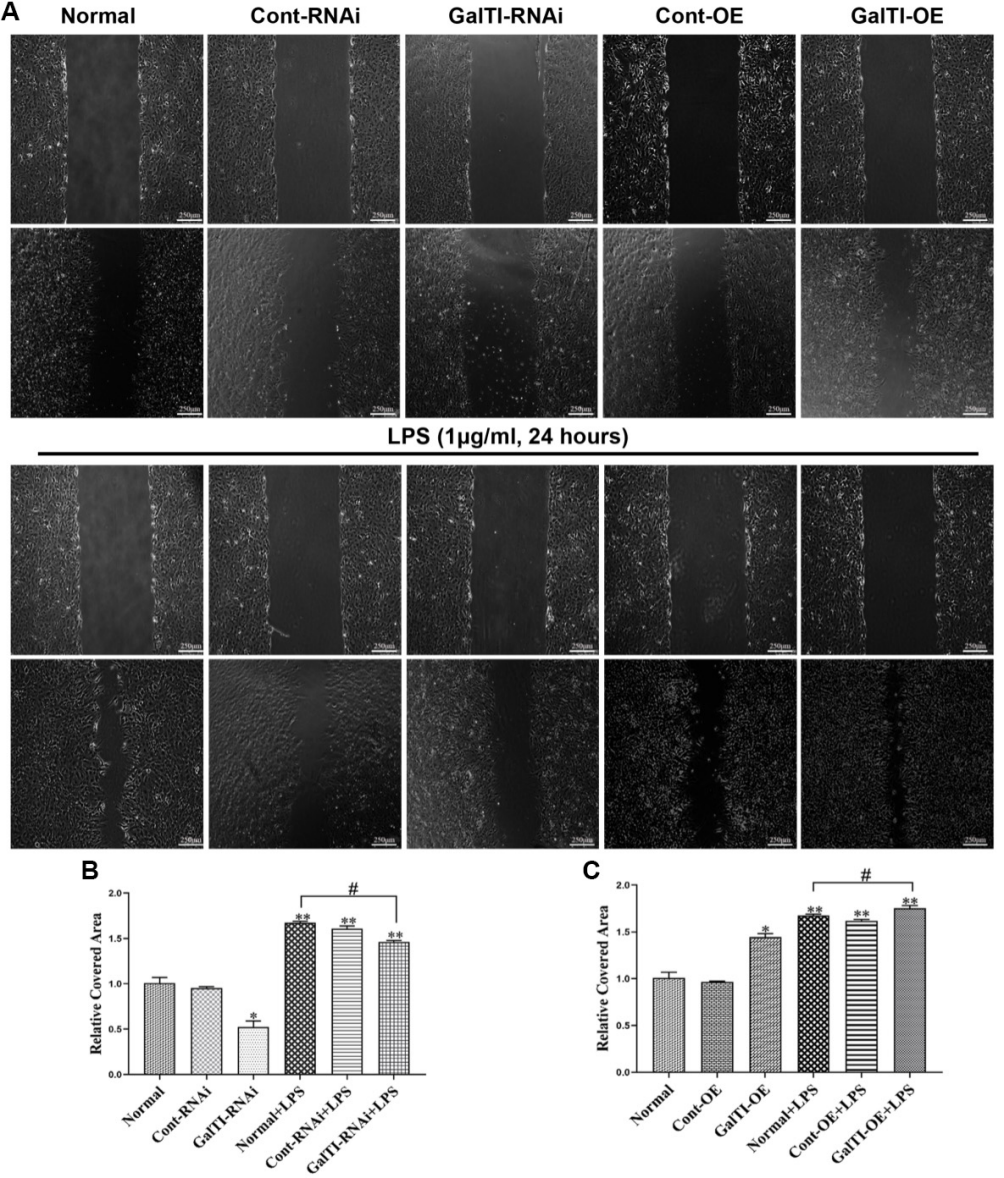
**Effects of *β-1,4-GalTI* intervention on the migration of LPS-treated primary astrocytes.** (A) Migration of astrocytes in each group at 0 and 24 h; (B) and (C) Quantitative analysis of wound healing experiments; *n* ═ 3 per group, ^*^P < 0.05 vs Normal,^**^P < 0.01 vs Normal/Cont-RNAi. Normal/Cont-RNAi /GalTI-RNAi/Cont-OE/GalTIOE, ^#^*P* < 0.05 vs Normal+LPS; Bar ═ 250 µm. LPS: Lipopolysaccharide.

The Transwell migration experiment demonstrated that the interference of *β-1,4-GalTI* in astrocytes led to an increase in their migration capacity. This increase was observed in the Normal+LPS group, Cont-RNAi+LPS group, and GalTI-RNAi+LPS group when compared to the Normal group, Cont-RNAi group, and GalTI-RNAi group, respectively (all *P* < 0.05). In comparison to the Normal group, the GalTI-RNAi group showed a significant decrease in the migration capacity of astrocytes (*P* < 0.05). Similarly, the GalTI-RNAi+LPS group exhibited a decrease in astrocyte migration compared to the Normal+LPS group (*P* < 0.05). These findings suggest that interfering with *β-1,4-GalTI* can inhibit astrocyte migration (Figure 6). At the same time, the results of the Transwell migration experiment revealed that the group with overexpression of *β-1,4-GalTI* exhibited enhanced migration capacity of astrocytes compared to the Normal group, Cont-OE group, and GalTI-OE group. Specifically, the Normal+LPS group, Cont-OE+LPS group, and GalTI-OE+LPS group demonstrated a significant increase in astrocyte migration (all *P* < 0.05). These findings suggest that LPS plays a role in promoting astrocyte migration. Additionally, the migration capacity of astrocytes was found to be higher in the GalTI-OE group compared to the Normal group (*P* < 0.05). Similarly, the GalTI-OE+LPS group showed an increase in astrocyte migration compared to the Normal+LPS group (*P* < 0.05). These findings suggest that overexpression of *β-1,4-GalTI* can enhance astrocyte migration (Figure 6). Similarly, after astrocytic lentivirus treatment, the effect of cell migration was examined using a scratch assay. The cell scratch assay results revealed that, compared to the control groups (Normal, Cont-RNAi, GalTI-RNAi, Cont-OE, and GalTI-OE), the addition of LPS significantly increased the relative coverage area of astrocytes in the Normal+LPS, Cont-RNAi+LPS, GalTI-RNAi+LPS, Cont-OE+LPS, and GalTI-OE+LPS groups (*P* < 0.05 for all), demonstrating LPS’s role in enhancing astrocyte migration. Specifically, the GalTI-RNAi group exhibited a reduction in cell coverage area compared to the Normal group (*P* < 0.05), whereas the GalTI-OE group showed an increase (*P* < 0.05). Furthermore, relative to the Normal+LPS group, cell coverage was reduced in the GalTI-RNAi+LPS group (*P* < 0.05) but increased in the GalTI-OE+LPS group (*P* < 0.05). These results indicate that interference with *β-1,4-GalTI* inhibits, while its overexpression promotes, astrocyte migration. The results of the cell scratch assay are consistent with the Transwell migration experiment (Figure 7).

### Involvement of β*-1,4-GalTI* in the release of TNF-α, IL-1β, and IL-6 stimulated by LPS

TNF-α, IL-1β, and IL-6 are typical pro-inflammatory cytokines that play crucial roles in immune modulation and the neuroinflammatory process [36]. Moreover, TNF-α and IL-1β also play a role in astrocyte proliferation [37]. Previous research demonstrated that when primary astrocytes were treated with 1 µg/mL LPS for 24 h, there was a significant increase in the expression levels of pro-inflammatory cytokines TNF-α, IL-1β, and IL-6 [38]. In this study, we aimed to investigate the impact of intervening *β-1,4-GalTI* on the inflammatory response of astrocytes. To achieve this, astrocytes were initially transfected with interfering and overexpressing *β-1,4-GalTI* lentiviral vectors for 48 h. Subsequently, astrocytes in each group were exposed to 1 µg/mL LPS for 24 h. The release of inflammatory factors TNF-α, IL-1β, and IL-6 in the cells of each group was then measured using ELISA kits.

**Figure 8.**
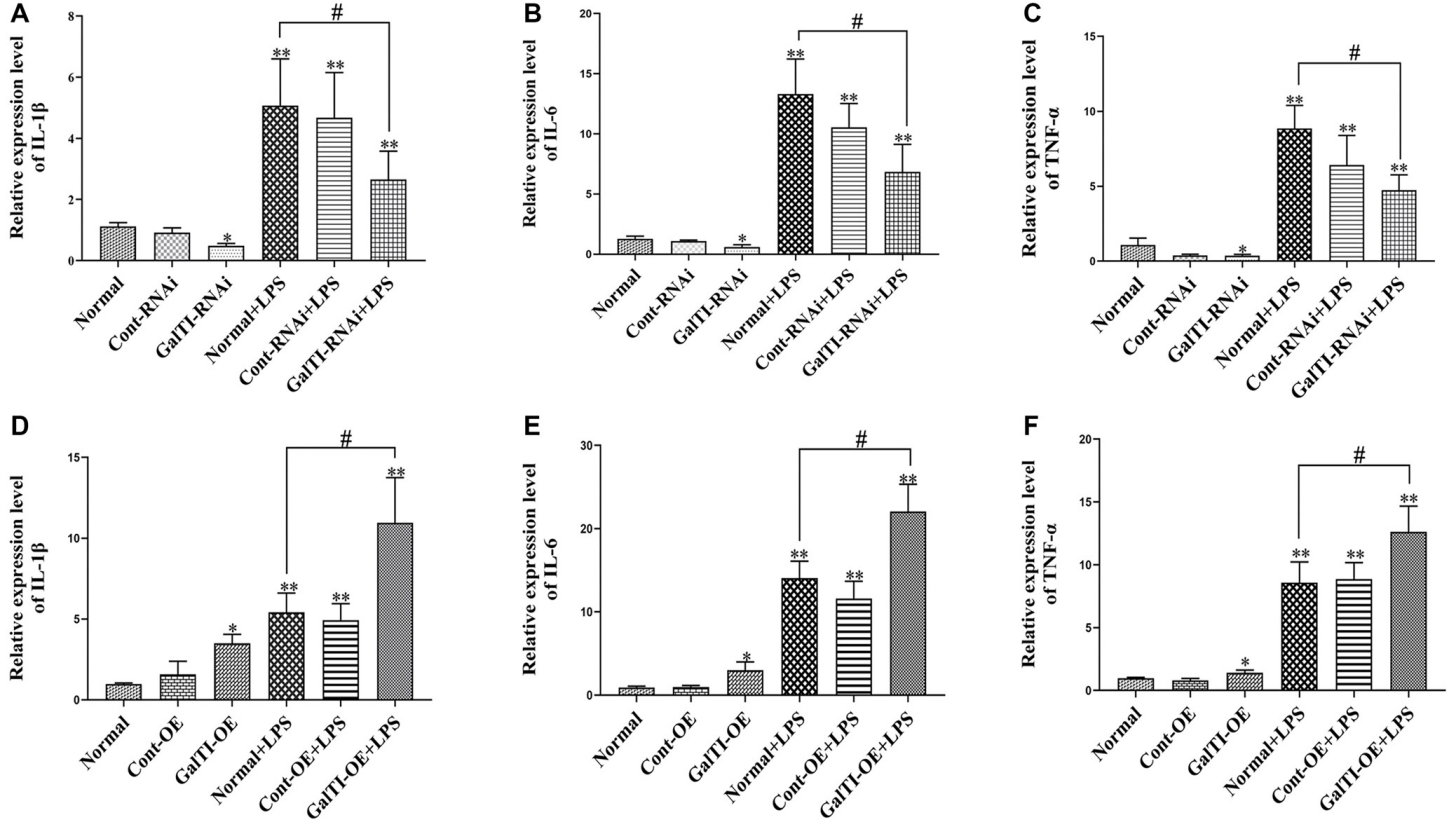
**Involvement of *β-1,4-GalTI* in release of IL-1β, IL-6, and TNF-α stimulated by LPS.** (A)–(C) The expressions of pro-inflammatory cytokines IL-1-β, IL-6, and TNF-α in the *β-1,4-GalTI* interference group were detected by ELISA; (D)–(F) The expressions of pro-inflammatory cytokines IL-1-β, IL-6, and TNF-α in the *β-1,4-GalTI* overexpression group were detected by ELISA; *n* ═ 3 per group, ^*^P < 0.05 vs Normal,**P < 0.01 vs Normal/Cont-RNAi/GalTI-RNAi/Cont-OE/GalTI-OE,^#^*P* < 0.05 vs Normal + LPS. IL-1β: Interleukin-1β; IL-6: Interleukin-6; TNF-α: Tumor necrosis factor-α; LPS: Lipopolysaccharide; ELISA: Enzyme-linked immunosorbent assay.

Compared to the Normal group, Cont-RNAi group, and GalTI-RNAi group, the ELISA test results of the *β-1,4-GalTI* interference group showed a significant elevation (all *P* < 0.05) in the release amounts of TNF-α, IL-1β, and IL-6 in the Normal+LPS group, Cont-RNAi+LPS group, and GalTI-RNAi+LPS group. This indicates that LPS stimulates the release of inflammatory factors, such as TNF-α, IL-1β, and IL-6. In comparison to the Normal group, the GalTI-RNAi group exhibited decreased levels of TNF-α, IL-1β, and IL-6 release (all *P* < 0.05). Similarly, the GalTI-RNAi+LPS group showed reduced release of TNF-α, IL-1β, and IL-6 compared to the Normal+LPS group (all *P* < 0.05). These findings suggest that interference with *β-1,4-GalTI* can effectively inhibit the release of inflammatory factors TNF-α, IL-1β, and IL-6 (Figure 8A–8C). However, compared to the Normal group, Cont-OE group, and GalTI-OE group, the ELISA test results of the *β-1,4-GalTI* overexpression group showed a significant increase in the release amounts of TNF-α, IL-1β, and IL-6 in the Normal+LPS group, Cont-OE+LPS group, and GalTI-OE+LPS group (all *P* < 0.05). This suggests that LPS enhances the release of inflammatory factors, such as TNF-α, IL-1β, and IL-6. In comparison to the Normal group, the GalTI-OE group exhibited elevated levels of TNF-α, IL-1β, and IL-6 release (all *P* < 0.05). Similarly, the GalTI-OE+LPS group showed higher levels of release compared to the Normal+LPS group. The overexpression of *β-1,4-GalTI* significantly elevated the levels of TNF-α, IL-1β, and IL-6 (*P* < 0.05), indicating its ability to stimulate the production of these inflammatory cytokines (Figure 8D–8F).

## Discussion

*β-1,4-GalTI* is widely and strongly expressed in almost all tissues and acts as a cell surface adhesion molecule. It mediates a variety of cell–cell and cell–matrix interactions [39], such as sperm–egg binding, cell adhesion and migration [40–42], tumorigenesis and progression, and neurite outgrowth. It has been studied in astrocytes, endothelial cells, immune cells, and tumor cells [43–45]. Additionally, research has demonstrated that *β-1,4-GalTI* plays a significant role in the inflammatory processes initiated by microglia during CNS inflammatory responses. Although there is extensive research on *β-1,4-GalTI,* studies specifically addressing its role in astrocytes remain limited.

In this study, we confirmed that *β-1,4-GalTI* is expressed in astrocytes and significantly influences astrocyte migration and proliferation during both quiescent and LPS-triggered activated phases. Additionally, manipulating β-1,4-GalTI expression exerts a regulatory effect on the release of cytokines, such as IL-1β, TNF-α, and IL-6.

To further study the effect of *β-1,4-GalTI* on the biological function of LPS-treated astrocytes, we constructed an LPS-induced cellular inflammation model based on *β-1,4-GalTI*. Interference and overexpression strategies were used to explore the possible pathways of *β-1,4-GalTI* affecting astrocyte inflammation. We first detected the purity of astrocytes in the cultured primary SD rat cerebral cortex using cell immunofluorescence. The results showed that the cell purity was over 90%, which could be used for subsequent studies.

Next, we used immunofluorescence staining to detect whether *β-1,4-GalTI* was expressed in primary astrocytes. The results showed that astrocytes in the cerebral cortex of SD rats *β-1,4-GalTI* co-localized with the astrocyte marker GFAP and the nuclear marker Hoechst33342, indicating that *β-1,4-GalTI* was present in both the cytoplasm and nucleus of astrocytes.

Lentiviral vectors are gene therapy vectors developed from the human immunodeficiency virus, which can effectively integrate foreign genes into the host chromosome and are a commonly used vector. They have the advantages of high transfection efficiency, stable expression, large accommodation for exogenous target gene fragments, and the ability to infect both dividing and non-dividing cells, with low cytotoxicity and immunogenicity. They are often used to infect neurons, endothelial cells, stem cells, etc. The *β-1,4-GalTI* interference and overexpression lentiviral vectors used in this study were purchased from Shanghai Jikai Gene Company. We analyzed the efficiency of lentivirus transfection into SD rat primary astrocytes by cell immunofluorescence to determine whether the lentiviral vector was successfully constructed. We first explored the *β-1,4-GalTI* lentivirus infection coefficient (Multiplicity of Infection [MOI]), and the results showed that the transfection efficiency of each group of lentiviruses was above 85% at MOI ═ 20. Considering both research cost and results, we selected MOI ═ 20 for the follow-up research. Through qRT-PCR screening, 570-1 was the most interfering lentiviral vector, while 68233-1 was an effective lentiviral overexpression vector. Therefore, primary astrocytes were transfected with the 570-1 lentiviral interference vector and the 68233-1 lentiviral overexpression vector in subsequent studies. The expression of *β-1,4-GalTI* was assessed in the normal group of transfected cells and non-lentivirus-transfected cells from each group using PCR. This analysis revealed a decrease in *β-1,4-GalTI* expression in the normal group, while expression increased in the GalTI-OE group, confirming the successful construction of the lentiviral vectors. Concurrently, Western blot analysis was conducted to measure *β-1,4-GalTI* levels in cells from each group, and the results were consistent with those obtained from PCR.

Astrocytes respond to CNS injuries by proliferating and migrating toward the injury site, culminating in the formation of glial scars. This reactive glial proliferation mechanism impedes axonal regeneration [33, 46]. Moreover, inflammatory responses can promote astrocyte migration and exacerbate glial scar formation [47]. Research has indicated that *β-1,4-GalTI* may play a crucial role in regulating the migration of immune cells to inflammation sites. Substrates of Src-inhibited protein kinase C affect astrocyte migration and the galactosylation of integrin β1 by functionally modulating *β-1,4-GalTI* catalysis in LPS-induced astrocyte inflammation [47]. In this study, CCK-8 and EdU proliferation assays showed that LPS promoted astrocyte proliferation, *β-1,4-GalTI* interference inhibited astrocyte proliferation, and *β-1,4-GalTI* overexpression promotes the proliferation of astrocytes. The results of Transwell^TM^ migration assays and cell scratch assays showed that LPS promoted astrocyte migration, *β-1,4-GalTI* interference inhibited astrocyte migration, and *β-1,4-GalTI* overexpression promoted astrocyte migration. LPS-stimulated astrocytes can express various cytokines, such as TNF-α, IL-1β, and IL-6, as well as chemokines like CCL2, CXCL1, CCL20, and CCL3. Among them, TNF-α, IL-1β, and IL-6 are the most typical and studied pro-inflammatory cytokines, playing important roles in immune regulation and neuroinflammation [48]. In the nervous system, LPS can induce neuroinflammation and mimic the inflammatory response following spinal cord injury. LPS-induced chronic inflammation can stimulate the production of pro-inflammatory cytokines and the deposition of amyloid β-protein, mimicking some of the neurodegenerative processes involved in AD [49]. Additionally, LPS can mimic Parkinson’s disease by inducing inflammation in the substantia nigra, leading to degeneration of dopaminergic neurons. LPS can also mimic the immune-mediated myelin attack seen in multiple sclerosis (MS) by inducing microglia activation and releasing inflammatory mediators [50]. By inducing systemic or localized inflammation, LPS has helped researchers explore many other neuroinflammation-related diseases, such as amyotrophic lateral sclerosis (ALS), sepsis-associated encephalopathy (SAE), neuropathic chronic pain, Huntington’s disease (HD), and stroke [51–53]. *β-1,4-GalTI* serves as a critical inflammatory mediator and plays a pivotal role in the initiation and progression of numerous inflammatory diseases [54]. Therefore, we believe that intervening the expression of *β-1,4-GalTI* in astrocytes may aid in searching for treatments for neuroinflammation-related diseases. Research has shown that LPS influences the expression of *β-1,4-GalTI* in Schwann cells and microglia in a time- and concentration-dependent manner, with increased expression potentially linked to the inflammatory process and TNF-α secretion. Mice deficient in the *β-1,4-GalTI* gene exhibit impaired leukocyte infiltration and selectin ligand biosynthesis, leading to attenuated acute and chronic inflammatory responses. Initial investigations by our research team revealed that exposing primary astrocytes to 1 µg/mL of LPS for 24 h resulted in elevated expression levels of pro-inflammatory proteins TNF-α, IL-1β, and IL-6 [38]. Thus, in this investigation, the objective was to clarify the effect of intervening with *β-1,4-GalTI* on astrocyte inflammatory responses. To do this, primary astrocytes were initially transfected with interfering and overexpressing *β-1,4-GalTI* lentiviral vectors for 48 h. Subsequently, they were treated with a concentration of 1 µg/mL of LPS for 24 h. The release of pro-inflammatory molecules TNF-α, IL-1β, and IL-6 from cells in each group was detected using ELISA kits. The findings demonstrated that following LPS stimulation of astrocytes, the expression levels of pro-inflammatory factors TNF-α, IL-1β, and IL-6 were increased. The interference of *β-1,4-GalTI* suppressed the expression of pro-inflammatory factors TNF-α, IL-1β, and IL-6, whereas the overexpression of *β-1,4-GalTI* enhanced the expression of pro-inflammatory factors TNF-α, IL-1β, and IL-6.

## Conclusion

In conclusion, our work revealed that interfering with *β-1,4-GalTI* had a specific effect on the growth, migration, and secretion of pro-inflammatory cytokines from astrocytes treated with LPS. These findings indicate that *β-1,4-GalTI* could be a promising target for addressing astrocyte inflammation. However, there is a gap in understanding the specific mechanisms by which *β-1,4-GalTI* influences astrocyte physiology and its direct interactions with other cellular components in the CNS. Therefore, in the future, we will conduct experiments to investigate the role of *β-1,4-GalTI* in CNS inflammation or injury in vivo. Additionally, we will explore its interactions with neurons to investigate the neuroprotective role of *β-1,4-GalTI* in astrocytes.

## Data Availability

The data that supports the findings of this study is available upon a reasonable request from the corresponding and first authors.
